# Field-Based Scoring of Soybean Iron Deficiency Chlorosis Using RGB Imaging and Statistical Learning

**DOI:** 10.3389/fpls.2018.01002

**Published:** 2018-07-11

**Authors:** Geng Bai, Shawn Jenkins, Wenan Yuan, George L. Graef, Yufeng Ge

**Affiliations:** ^1^Department of Biological Systems Engineering, University of Nebraska–Lincoln, Lincoln, NE, United States; ^2^Department of Agronomy and Horticulture, University of Nebraska–Lincoln, Lincoln, NE, United States

**Keywords:** abiotic stress, high throughput phenotyping, image processing, linear discriminant analysis, support vector machine

## Abstract

Iron deficiency chlorosis (IDC) is an abiotic stress in soybean that can cause significant biomass and yield reduction. IDC is characterized by stunted growth and yellowing and interveinal chlorosis of early trifoliate leaves. Scoring IDC severity in the field is conventionally done by visual assessment. The goal of this study was to investigate the usefulness of Red Green Blue (RGB) images of soybean plots captured under the field condition for IDC scoring. A total of 64 soybean lines with four replicates were planted in 6 fields over 2 years. Visual scoring (referred to as Field Score, or FS) was conducted at V3–V4 growth stage; and concurrently RGB images of the field plots were recorded with a high-throughput field phenotyping platform. A second set of IDC scores was done on the plot images (displayed on a computer screen) consistently by one person in the office (referred to as Office Score, or OS). Plot images were then processed to remove weeds and extract six color features, which were used to train computer-based IDC scoring models (referred to as Computer Score, or CS) using linear discriminant analysis (LDA) and support vector machine (SVM). The results showed that, in the fields where severe IDC symptoms were present, FS and OS were strongly positively correlated with each other, and both of them were strongly negatively correlated with yield. CS could satisfactorily predict IDC scores when evaluated using FS and OS as the reference (overall classification accuracy > 81%). SVM models appeared to outperform LDA models; and the SVM model trained to predict IDC OS gave the highest prediction accuracy. It was anticipated that coupling RGB imaging from the high-throughput field phenotyping platform with real-time image processing and IDC CS models would lead to a more rapid, cost-effective, and objective scoring pipeline for soybean IDC field screening and breeding.

## Introduction

Iron deficiency chlorosis (IDC) is a serious abiotic stress in soybean characterized by stunted growth, yellowing and interveinal chlorosis of the early trifoliate leaves (Froechlich and Fehr, 1981). Yield losses have been estimated in excess of $120 million annually in the western Corn Belt and Great Plains regions of the United States (Hansen et al., 2004). IDC is directly related to the inability of soybean plants to uptake iron (Fe) due to low soil Fe solubility. With reduced Fe uptake, photosynthesis of soybeans is drastically limited (Mengel, 1994; Jiang et al., 2007; Vasconcelos and Grusak, 2014), which further causes the decrease in desirable characteristics such as biomass and yield (Mengel, 1994; Jiang et al., 2007; Briat et al., 2015).

Grain yield can be significantly impacted if visual symptoms of IDC are present during the growing season. Visual scoring is a key tool for assessing the variation of soybean plants in IDC tolerance (Niebur and Fehr, 1981; Inskeep and Bloom, 1987). The visual IDC symptoms of soybean plants are more pronounced in calcareous soils with higher pH (> 8.0) (Froechlich and Fehr, 1981; Jessen et al., 1988; Goos and Johnson, 2000; Hansen et al., 2004). Although a number of methods were investigated to reduce the economic impact of IDC, planting tolerant cultivars remains the most cost-effective approach to address the negative effect of IDC (Goos and Johnson, 2000). Visual scoring of IDC on soybean cultivars to be released is of great importance to producers, because of the high correlation of IDC symptoms to yield.

Traditionally, visual scoring of IDC severity is carried out manually by trained researchers when symptoms are maximally expressed at approximately V3 growth stage (Fehr et al., 1971; Rodriguez de Cianzio et al., 1979; Froechlich and Fehr, 1981). The speed of IDC visual scoring can be a limitation when a large number of plots are to be assessed. With the development of new node occurring approximately every 4 days during the vegetative growth stages, logistic difficulties related to timing and labor availability can arise to score the IDC severity when maximally expressed (Bastidas et al., 2008). Furthermore, human inconsistency would almost inevitably be introduced in this process due to the fluctuation in field illumination conditions (e.g., sunny vs. overshadowed days), physical condition of the researchers, and variation in color perception among researchers.

More recently high-throughput plant phenotyping (HTPP) has emerged as a new research frontier to relieve the bottleneck of plant phenotyping, accelerate plant breeding, and use plant genomic data more effectively (Furbank and Tester, 2011; Fiorani and Schurr, 2013; Araus and Cairns, 2014). A lot of work has been done to develop imaging and sensing platforms that can rapidly measure plant morphological and physiological traits in the field (Andrade-Sanchez et al., 2014; Sankaran et al., 2015; Virlet et al., 2017). The emphasis is mainly on plant breeding programs to evaluate many genetic lines repeatedly in a season by using multiple imaging and sensing modalities. IDC changes the color of soybean leaves substantially in a specific growth stage. Conceivably, Red Green Blue (RGB) images of soybean plots can be captured by an HTPP system. These images can further be processed to extract color information of the leaves and to develop automated scoring algorithm for IDC severity.

Naik et al. (2017) and Zhang et al. (2017) used a CANON digital camera to take RGB images of soybean plants in the field. The authors further tested a number of classification methods to score IDC severity using color features extracted from the images. They showed that manual IDC scores were satisfactorily predicted from image color features, which suggested the great potential to develop an imaging-based, automated IDC scoring system. A great deal of effort, however, was reported in this study for image acquisition, calibration, and weed exclusion – which could substantially lower the analysis throughput. In addition, their scoring was done on single plants, whereas in practice, a group of plants from a field plot is scored.

The objectives of this study were to (1) use an existing HTPP sensor platform to collect RGB images from soybean IDC field trials; (2) develop an automated, computer-based scoring of IDC using RGB images; and (3) compare the effectiveness of Computer Scoring (CS) of IDC to the conventional manual scoring. To the best of our knowledge, this is the first attempt to employ HTPP in field-based scoring of soybean IDC with the aim to increase the analysis throughput and objectivity for whole-plot scoring.

## Materials and Methods

### Experiment Design and Field Management

The field experiment was conducted for 2 years near Fremont and North Bend, NE, United States. Six fields (two in 2016 and four in 2017) were selected using soil pH and historical soybean IDC response as the criteria, with the goal to create contrasting IDC severity among these fields for phenotyping. A total of 10–15 soil samples were collected from each field and analyzed in a commercial laboratory for pH and available Iron.

In each field, soybeans were planted as 2.9-m-long two-row plots. The row spacing was 0.762 m. The space between plots was 0.9 m to serve as alleyway for manual IDC scoring and plot harvest. Two sets of plant material were evaluated. The first group had 21 entries, consisting of 11 experimental lines developed by the University of Nebraska Soybean Breeding program through 10 cycles of recurrent selection for improved IDC tolerance, and 10 advanced experimental lines representing diversity in IDC response (Kocak, 2014). The second group contained 43 entries, consisting of two commercial check cultivars, two parental lines of a recombinant inbred line (RIL) population developed for differing IDC responses, and 39 F6:8 experimental lines selected for extremes in IDC responses from a large RIL population developed by the University of Nebraska Soybean breeding program (Kocak, 2014). Each test was designed as a randomized complete block design (RCBD) with test serving as blocks and four block replicates, therefore giving 256 plots [(21 + 43) × 4] in each field. A total of 1530 plots (256 × 6–6) were scored for the six fields across the 2 years; and the missing six plots were due to poor plot establishment.

**Table 1** summarizes the information of the field experiment including soil type, plot number, and dates of visual scoring and phenotyping data collection, planting, and harvest. The plots were dried to uniform moisture content and harvested with a two-row Almaco SPC-40 plot combine. Yield data were adjusted to 13% moisture content. Yield data were not available for Field 6 in 2017 North Bend, due to the severe IDC symptoms in combination with a devastating hail occurred shortly after IDC scoring (Jun/30).

**Table 1:** The information regarding the soybean iron deficiency chlorosis field phenotyping experiment.

Year–Location	Date of visual scoring | image collection	Planting | harvesting date	Soil type	Field	Plot number
2016–Fremont	Jul/7 | Jul/12	Jun/9 | Oct/20	Gibbon-Wann Complex, silt loam	1	256
				2	254
2017–Fremont	Jun/27 | Jun/27	Jun/3 | Nov/2	Saltine-Gibbon complex silty clay loam	3	256
				4	256
2017–North Bend	Jun/27 | Jun/27	Jun/3 | Nov/2	Saltine-Gibbon complex silty clay loam	5	254
				6	254

### Field-Based Manual Scoring of IDC Severity

**FIGURE 1:**
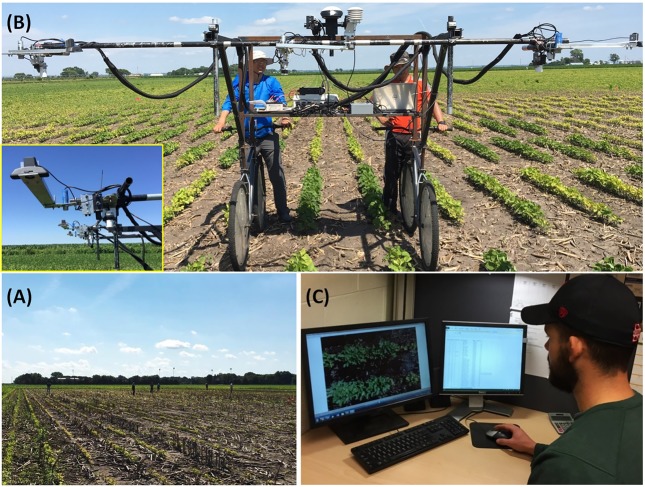
Soybean field experiment data collection and iron deficiency chlorosis severity scoring. **(A)** Field scoring by a group of 5–6 trained researchers; **(B)** RGB image acquisition of field plots with a high-throughput field phenotyping platform. The inset shows the webcam used for image acquisition; **(C)** Office scoring in front of a computer monitor by SJ (the second author of this paper).

Field scoring (FS) of IDC was conducted manually when the soybean plots were at V3–V4 developmental stage. A 9-point scale was employed to score the severity of IDC symptom, with a score of 1 = no yellowing; 3 = mild yellowing; 5 = moderate interveinal chlorosis; 7 = severe interveinal chlorosis; and 9 = dead meristems or plants. In each field, a group of 5–6 trained researchers participated in field scoring (**Figure 1A**). Every scorer was trained in the following fashion. First, soybean images that illustrated IDC scores of 1, 3, 5, 7, and 9 were distributed to each scorer. They spent time to become familiar with those as shown in the images before scoring. Then, several plots in the field were scored by all scorers in order to calibrate the scores given by different scorers. During the scoring, each scorer rated an entire block to eliminate the between-scorer variation within a block in the field. It should be noted that FS could still introduce scorer-specific bias into the field score because multiple scorers were involved in the process across different site-years, even though every scorer was appropriately trained. Another round of visual rating was conducted by a designated researcher using the plot images captured by the field phenotyping platform (see the section “Plot Image Collection With a High-Throughput Plant Phenotyping Platform and Office Scoring of IDC” below) in the office (referred to as office scoring, or OS).

### Plot Image Collection With a High-Throughput Plant Phenotyping Platform and Office Scoring of IDC

The HTPP platform used in this study was previously developed and reported in Bai et al. (2016). The platform was able to collect an array of plant phenotyping data (canopy temperature, height, reflectance spectra, normalized difference vegetation index, and RGB image) and environmental data (air temperature, relative humidity, and shortwave solar radiation) from three two-row plots simultaneously (**Figure 1B**). In this study, only the RGB images from soybean plots were used for IDC scoring. The cameras were low-cost webcams (Logitech C920). They were placed at ∼1.55 m above the soybean canopy such that the camera’s field of view matched the width of the plots. The cameras were also oriented and adjusted such that the crop row was in parallel with the *X*-axis of the image and the two crop rows were roughly centered at the *Y*-axis. The images were 2304 × 1536 in pixels with a resolution of 0.9 mm/pixel. A fixed brightness value for the cameras was determined before each imaging campaign to avoid over or under exposure. This method worked well and maintained good color consistency of soybean images, due to the following two factors. First, all images were taken under cloudless, fully sunny sky condition. Second, the measurement speed of the HTPP platform (∼0.2 ha/h) allowed us to limit the image collection time window between 11:00 AM and 3:00 PM (local solar noon around 1:30 PM). These conditions minimized the color inconsistency of images due to clouds and low solar angles.

**FIGURE 2:**
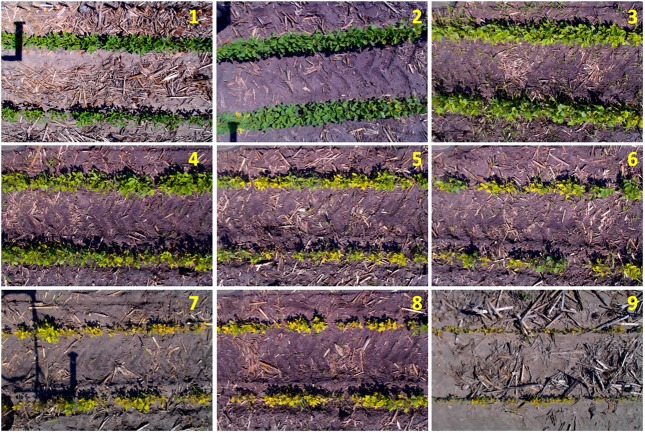
Example RGB images of soybean field plots (captured by the high throughput field phenotyping platform) that were scored IDC 1–9 consistently by both field score and office score.

After plot images were acquired and stored, a second set of IDC scores was made by displaying the images on a computer screen and scored by the designated researcher (SJ, the second author of this paper), using the same 9-point scale in the field (**Figure 1C**). This set of scores was referred to as Office Score (OS). One major interest was to examine the relationship between OS and FS. **Figure 2** gives the example RGB images of soybean field plots that were scored IDC 1 through 9 consistently by both FS and OS.

### Digital Image Processing

The raw RGB images of soybean plots were processed in order to extract relevant parameters for automated, computer-based IDC scoring. The following steps were applied. First, the raw image was converted from RGB to Hue Saturation Value (HSV) color space; and a Hue value from 40 to 170°, which covered green, yellow, and orange color range, was used to carry out the initial plant pixel segmentation. This approach was different from many other studies where certain green indices were used to segment green plant pixels from background (Deery et al., 2014). Because soybean with moderate to severe IDC symptoms usually have substantial leaf yellowing or browning, focusing on just the green pixels could not segment all soybean plants. A hue value from 40 to 170° was wide enough to cover the color variation of healthy and stressed plants, and also effectively excluded soil background. Second, a morphological opening operation was applied to remove isolated noise from the segmented binary images.

**FIGURE 3:**
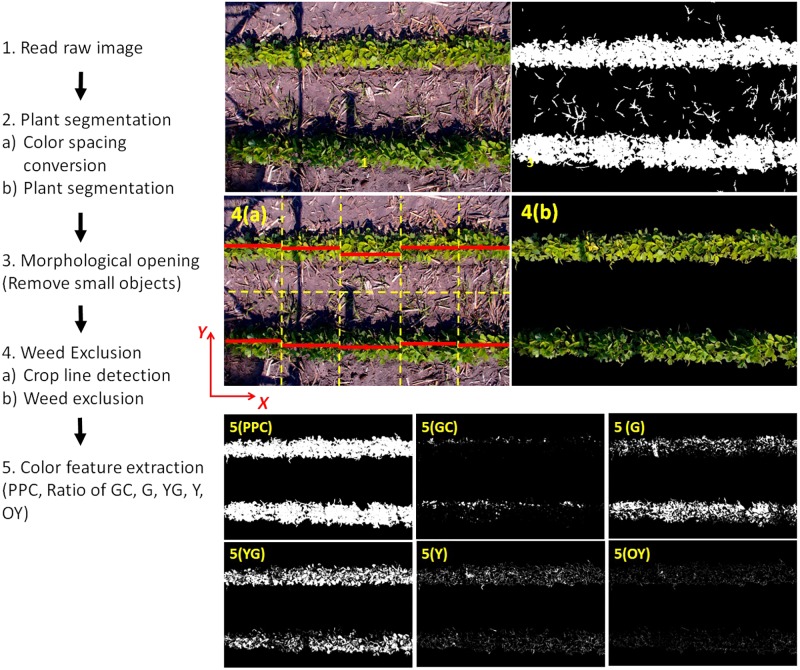
Custom algorithm to process RGB images of soybean plots, exclude weeds, and extract plant color parameters for subsequent statistical modeling.

Weeds were present in a large portion of the images. Because weeds were green and large in size (compared to isolated noise), they could not be effectively removed by the segmentation or morphological operation. A custom algorithm was therefore developed for weed removal, whose work flow was given in detail below and also illustrated in **Figure 3**.

The two-row images were first divided into two one-row images by horizontally cutting the image into half. Because the crop line might not be perfectly in parallel to the *X*-axis, the one-row image was divided into five vertical sections (orange dash lines) and the crop line (red stripe) in each section was located. This was achieved by summing the plant pixels along the *X*-direction and the crop line was considered to be at the *Y*-position with the maximum plant pixel counts. There was a possibility that multiple maximum counts were found because the plant pixels accounted 100% of the pixel along the *X*-axis at multiple *Y*-values. In this case, the *Y*-coordinates of first and the last max count were averaged as the *Y*-value of the crop line. All the coordinates of the crop line in the image were saved in a matrix for later use. After finding the crop lines, a thin line-shape mask was created based on the crop line information. Any plant pixel (after segmentation) was examined by the mask and only the plant pixels on the mask were retained as “center plant pixels”. Then, all the plant pixels connected to the “center plant pixels” were selected and all other plant pixels were excluded. This exclusion algorithm performed effectively to exclude any pixel which was not connected to the center of the crop lines. The result of weed exclusion was shown in **Figure 3** (4b).

Six parameters were extracted from the weed-excluded images. Plant Pixel Count (PPC) was the total count of plant pixels in the image representing the size of soybean plants. Plant Pixel Count Fraction (PPCF) was calculated by dividing PPC with the total pixel count of the image. Plant pixels were further categorized into five color classes using the Hue channel of the HSV color space: Green-Cyan class (Hue value between 140 and 170°), Green class (81 and 140°), Yellow-Green class (61 and 80°), Yellow class (51 and 60°), and Orange-Yellow class (41 and 50°). The fraction of each color class was then calculated as the color parameters, namely, Green-Cyan pixel fraction (Ratio_GC), Green pixel fraction (Ratio_G), Yellow-Green pixel fraction (Ratio_YG), Yellow pixel fraction (Ratio_Y), and Orange-Yellow pixel fraction (Ratio_OY). The sum of the above pixel ratios is 100% because the colors cover the entire range of the Hue channel segmented at Step 2.

Image processing was performed in MATLAB (Version R2016b) and the Image Processing Toolbox.

### Data Analysis and Automated Computer Scoring Models

Pearson’s correlation analysis was first conducted to relate IDC FS, IDC OS with yield in each field.

Two statistical learning methods, namely linear discriminant analysis (LDA) and support vector machine (SVM), were employed to develop automated, CS models to predict IDC ratings from the image parameters. The whole dataset (*n* = 1,530 by combining data from all 6 fields) was randomly and equally divided into 2 subsets, a training set and a test set. For each modeling method, two models were developed with either IDC FS or OS as the dependent variable. For SVM (using radial basis function kernel), a grid search of two tuning parameters (epsilon in insensitive loss function and cost of constraints violation) was performed, and the optimal SVM models were determined with the lowest Root Mean Squared Error of 10-fold cross validation.

The performance of the CS models was evaluated by applying them on the test set, and the overall accuracy rates of the classification were calculated and compared. Data analysis was performed in R environment (R Core Team, 2017) with package “e1071” (Meyer et al., 2017) for SVM modeling.

## Results

### Relationships Between IDC Field Score, Office Score, and Yield

The mean values of soil pH, available iron, soybean yield, IDC FS, and OS are summarized in **Table 2**. Soil pH in these fields ranged from 7.9 to 8.2, except for Field 5 with much lower pH of 7.2. Fields 3, 4, and 6 had soil available Fe lower than 10 ppm, whereas Fields 1 and 2 had soil available Fe higher than 10 ppm. Field 5 had a much higher soil available Fe than other fields (21.1 ppm).

**Table 2:** Field level summary statistics of relevant soil properties, soybean yield, and iron deficiency chlorosis scoring.

Field	Mean soil pH	Mean available iron (ppm)	Mean yield (Kg/ha)	Mean IDC field score and its range	Mean IDC office score and its range
1	7.9	11.4	3652	1.1 (1–2)	2.5 (1–7)
2	8.1	10.8	2562	2.3 (1–7)	4.1 (2–8)
3	8.2	8.3	3100	4.7 (1–9)	4.0 (1–8)
4	8.0	6.2	2663	4.8 (1–8)	4.5 (1–9)
5	7.2	21.1	2441	1.1 (1–5)	1.2 (1–4)
6	8.2	8.2	NA	6.8 (2–9)	5.3 (2–8)

There appeared to be a good correlation between the field-average IDC FS and IDC OS. Fields 3, 4, and 6 had both average FS and OS higher than 4.0, indicating quite severe IDC symptoms in these three fields. On the other hand, Fields 1 and 5 had average FS and OS lower than 2.5, indicating less IDC symptoms in these two fields. Field 2 had an average FS of 2.3 and average OS of 4.1. This field seemed to have an intermediate IDC stress among the six fields studied.

Comparisons between soil properties, IDC FS and OS, and soybean yield indicated the impact of soil pH and available Fe on soybean IDC severity and subsequently final yields. Data from Fields 1 and 2 were collected in 2016. When these two fields were compared, Field 2 had higher soil pH and lower available Fe. Both IDC FS and OS in Field 2 were higher than those of Field 1, and yield was appreciably lower. For the remaining four fields in 2017, Fields 3, 4, and 6 developed significant IDC symptoms. These fields had soil pH > = 8.0 and soil available Fe < 10 ppm. Field 4 showed a slightly more severe IDC symptoms than Field 3, as well as lower grain yield. Fields 5 and 6 (North Bend, 2017) underwent a hail damage, which stunted soybean growth severely and explained the low overall yield of Field 5 even with no apparent IDC symptoms. Whereas for Field 6, the combination of hail damage and severe IDC stress (highest FS and OS among the six fields studied) destroyed the majority of plots, making yield data not available.

**FIGURE 4:**
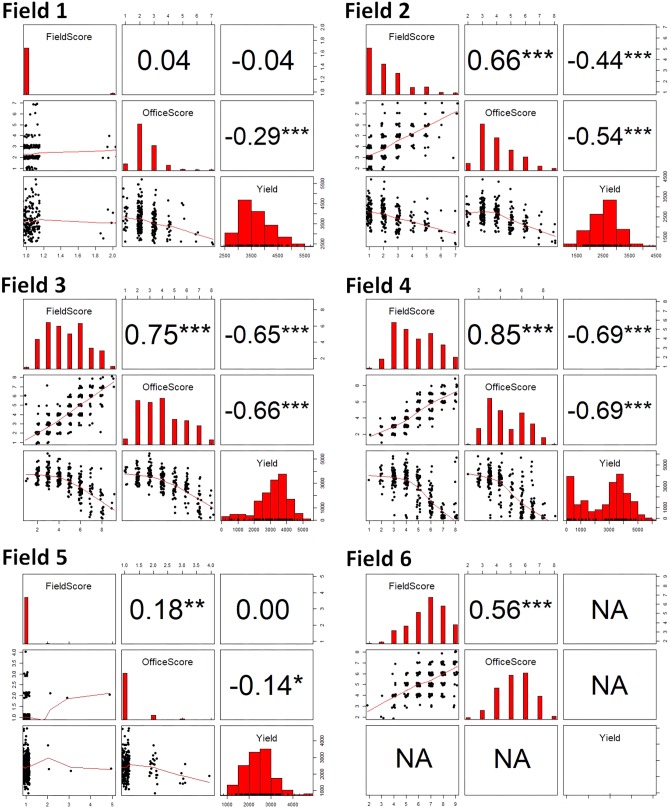
The scatterplots and Pearson’s correlation coefficients among iron deficiency chlorosis field score, office score, and yield for the six fields in this study. The significance levels of the Pearson’s correlation coefficients are: ^∗∗∗^ at 0.001 level, ^∗∗^ at 0.01 level, and ^∗^ at 0.05 level.

**Figure 4** shows the scatterplots and Pearson’s correlation coefficients among IDC FS, OS, and yield at the plot level for the six fields. It can be seen that IDC FS and OS were significantly correlated with each other in general. For Fields 2, 3, 4, and 6, the correlation coefficients between the two sets of IDC scoring ranged from 0.65 and 0.85 (*p* < 0.001). The correlation in Fields 1 and 5 were much weaker (*r* = 0.04 and 0.18, respectively). This was obviously due to the lack of IDC symptoms in these two fields. For instance in Field 1, FS only ranged between 1 and 2, with majority as 1; OS had a range between 1 and 7, with majority of them as 2 and 3. This narrow range in either IDC FS or OS was not wide enough to establish a stronger correlation. The general strong correlation between IDC FS and OS was expected, as this was the first hypothesis to be validated toward the use of imaging and statistical learning for automated IDC rating.

For the fields that showed obvious IDC symptoms (Fields 2, 3, and 4; note yield in Field 6 was not recorded), similarly strong negative correlations were observed between IDC FS and yield, and between IDC OS and yield. For FS vs. yield, the correlation coefficients ranged between −0.44 and −0.69 (*p* < 0.001); and for OS vs. yield, the correlation coefficients ranged from −0.54 to −0.69 (*p* < 0.001). For Fields 1 and 5 where IDC symptoms were less severe, no correlation was observed between FS and yield. But there was weak correlation between OS and yield (*r* = −0.29 for Field 1 and *r* = −0.14 for Field 5, *p* < 0.05). A close examination of the scatterplots showed that OS resulted in higher IDC scores for a number of plots in these two fields, which gave rise to higher negative correlations between OS and yield.

### Relationship Between Image-Based Parameters and IDC Field Score and Office Score

**FIGURE 5:**
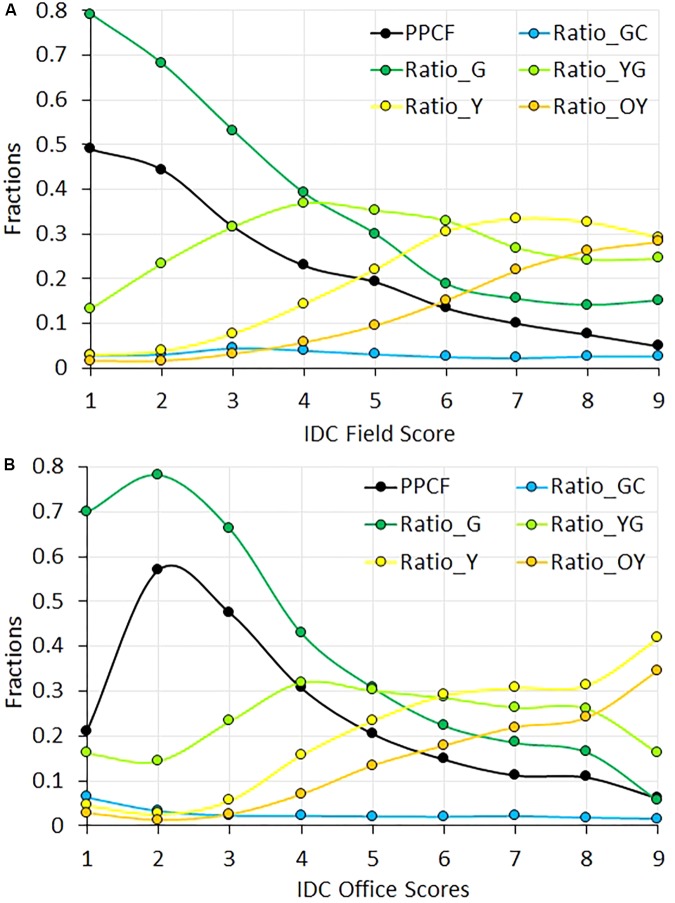
The trend of the six color parameters as a function of iron deficiency chlorosis (IDC) field score **(A)** and office score **(B)**. The color parameters were resulted from the processing of RGB images of soybean field plots.

Image processing described in the Section “Digital Image Processing” output six image-based parameters. The trends of these parameters as a function of IDC FS are given in **Figure 5A**. These parameters were categorized into three groups. PPCF and Ratio_G belonged to the first group, which exhibited sharp and consistent decrease as IDC scores increased. This indicated that the ground vegetation cover and green pixel declined quickly as IDC symptom became more severe (smaller plants and less healthy green vegetation). The second group included Ratio_YG, Ratio_Y, and Ratio_YO. These three parameters exhibited responses at various IDC scores. Ratio_YG increased from IDC FS 1 to 4, and then stayed more or less stable at higher IDC FS from 5 to 9. Ratio_Y was most sensitive between score 3 and 6 and showed saturation above score 7, whereas Ratio_OY was more responsive to higher IDC scores between 5 and 9. These trends corresponded well to the fact that as the IDC symptom became more severe, a higher proportion of soybean leaf areas turned from green to yellow to orange/brownish coloration. The sensitivity of these color parameters at different IDC ranges also suggested the potential advantage of using RGB images over visual scoring, as they may capture subtle color variations at different IDC stages. The third group included Ratio_GC, which remained low and changed little across the different IDC scores. Due to its insensitivity to IDC, Ratio_GC was not included in the statistical modeling for computer-based IDC scoring.

The trend of the image parameters with respective to IDC OS (**Figure 5B**) was similar to FS, with one difference. That is, the trend of PPCF and Ratio_G between IDC OS 1 and 2 were reversed compared to FS. A closer examination of FS vs. OS indicated that OS tended to give higher sores than FS for those plots having FS = 1. This trend suggested that PPCF might play a less important role for OS at lower IDC ranges than FS (in other words, OS relied more on color perception than plant size).

### Computer-Based IDC Scores

Using LDA, the overall accuracy of IDC CS was 55.0% (**Table 3**, Accuracy I) when evaluated against the actual FS in the validation set. Since a 9-point IDC scale was used, a random guess would have an average accuracy of 11.1%. Because a 5-point scale was also commonly used for IDC scoring (Goos and Johnson, 2000; Naik et al., 2017), Accuracy II was also calculated and reported by considering the cases where CS was only 1 point (orange cells in **Table 3**) off as accurate. Accuracy II was at a much higher 85% when CS from LDA method was compared to FS. When the CS was compared to OS, Accuracy I decreased to 45.0% and Accuracy II also decreased to 81.8%.

**Table 3:** Performance of soybean iron deficiency chlorosis scores predicted by the linear discriminant analysis model compared to the field score and office score (number of plots in validation: 1530/2 = 765).

	Field score										Office score									
		1	2	3	4	5	6	7	8	9		1	2	3	4	5	6	7	8	9
Computer Score	1	294	47	24	5	4	3	1	1	0	1	113	26	13	1	4	1	3	1	0
	2	0	0	0	0	0	0	0	0	0	2	10	70	47	12	0	0	0	0	0
	3	5	18	22	14	8	2	0	0	0	3	0	23	55	31	18	10	0	0	0
	4	0	7	24	23	9	1	1	0	0	4	1	9	38	41	12	12	5	1	0
	5	0	1	3	12	13	8	2	0	0	5	0	0	3	17	11	9	3	1	0
	6	0	1	3	14	23	27	16	5	1	6	0	0	2	15	23	35	14	4	0
	7	0	0	0	3	2	13	21	13	0	7	0	0	1	2	17	23	19	9	0
	8	0	0	0	0	2	8	18	18	8	8	0	0	0	0	0	0	0	0	0
	9	0	0	0	0	1	1	6	6	3	9	0	0	0	0	0	0	0	0	0
		Accuracy I = 55.0%										Accuracy I = 45.0%								
		Accuracy II = 85.0%										Accuracy II = 81.8%								

*Accuracy I was calculated using the blue cells from diagonal; Accuracy II was calculated using both blue and orange cells (meaning prediction was off by one point).*

The non-linear method SVM seemed to be more effective than the linear method LDA in modeling IDC FS and OS with the color features extracted from the RGB images (**Table 4**). When validated against IDC FS, CS SVM models yielded Accuracy I = 57.6% and Accuracy II = 87.7%. The Accuracy I and Aceloped and validated against IDC OS.

**Table 4:** Performance of soybean iron deficiency chlorosis scores predicted by the support vector machine model compared to the field score and office score (number of plots in validation: 1530/2 = 765).

	Field score										Office score									
		1	2	3	4	5	6	7	8	9		1	2	3	4	5	6	7	8	9
Computer Score	1	291	41	12	5	2	1	0	0	0	1	109	10	2	0	0	0	2	0	0
	2	1	0	0	0	0	0	0	0	0	2	15	79	25	3	1	0	0	1	0
	3	6	29	46	22	8	2	0	1	0	3	0	39	104	34	1	1	3	1	0
	4	1	2	14	19	11	3	2	0	0	4	0	0	26	55	27	18	1	1	0
	5	0	0	1	4	6	3	1	0	0	5	0	0	1	19	19	10	0	0	0
	6	0	2	3	17	29	33	17	4	1	6	0	0	1	6	32	46	19	3	0
	7	0	0	0	4	3	11	18	10	0	7	0	0	0	2	5	15	19	7	0
	8	0	0	0	0	3	10	27	28	11	8	0	0	0	0	0	0	0	0	0
	9	0	0	0	0	0	0	0	0	0	9	0	0	0	0	0	0	0	3	0
		Accuracy I = 57.6%										Accuracy I = 56.3%								
		Accuracy II = 87.7%										Accuracy II = 93.1%								

*Accuracy I was calculated using the blue cells from diagonal; Accuracy II was calculated using both blue and orange cells (meaning prediction was off by one point).*

## Discussion

Within each field, the average IDC FS and OS differed and such differences were not consistent across the six fields (**Table 2**). For Fields 1 and 2, average OS was higher than FS; whereas for Fields 3, 4, and 6, average OS was lower than FS. Because Fields 1 and 2 were from 2016 and Fields 3, 4, and 6 were from 2017, the reversed relationship between OS and FS was likely due to the inconsistency of either IDC scoring method between the years. OS was deemed more consistent because it was made by the designated researcher under the constant indoor environment. Because FS was done by different researchers in 2 years under different field conditions, one possible explanation was that IDC field scores were systematically lower in 2016 than 2017.

**FIGURE 6:**
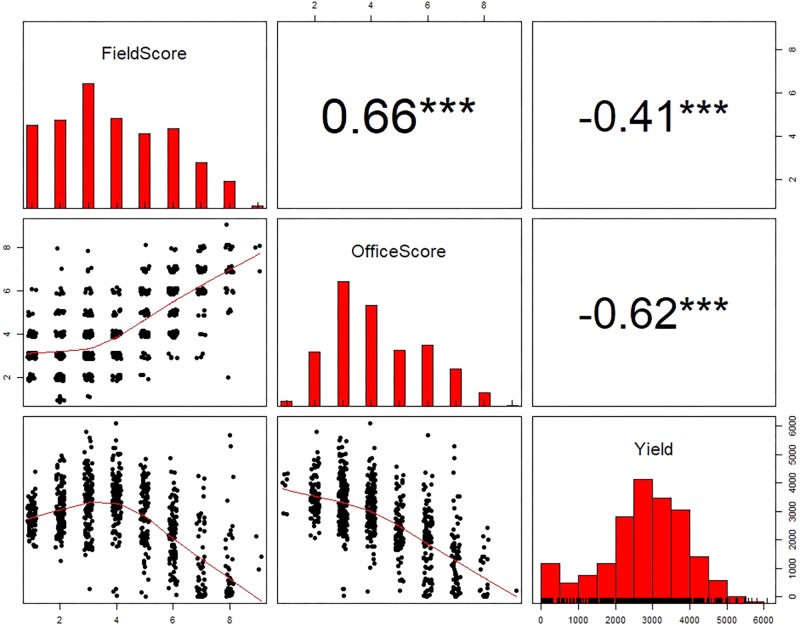
The scatterplots and Pearson’s correlation coefficients among iron deficiency chlorosis field score, office score, and yield for Fields 2, 3, and 4 combined. ^∗∗∗^ indicates the Pearson’s correlation coefficients are significant at 0.001 level.

To further explore the relationships between IDC FS, OS and yield, data from three fields (Fields 2, 3, and 4) were pooled and plotted in **Figure 6**. Note Fields 1 and 5 were excluded in this analysis because there were not adequate IDC symptoms, and Field 6 was excluded because yield was not recorded. The same correlation patterns were observed as compared to the individual fields. Strong and significant positive correlation existed between FS and OS (*r* = 0.66, *p* < 0.001). Strong and significant negative correlations between FS and yield and OS and yield were also observed for the pooled data. The difference, however, was that OS showed much stronger correlation with yield (*r* = −0.62) than FS (*r* = −0.41); whereas for individual fields, the degree of correlation was more comparable. This observation again supported the idea that OS could be a more consistent scoring system than FS. When the scatterplots in **Figure 6** were compared, yield vs. OS showed a consistent decrease; while yield vs. FS showed an apparent increase first between score 1 and 3, and then decreased from score 4 to 9. Since yield should be negatively correlated with IDC, this aberrant yield trend between FS 1 to 3 could be attributed to the inconsistency in FS among different fields. The inconsistency in FS was mainly caused by two factors. First, FS in different fields was made by different groups of researchers (**Figure 1A**) and OS was made by one researcher (**Figure 1C**). The difference in color perception and scoring among the observers could introduce systematic shift in FS from one field to another. Second, there were significant fluctuations in the natural light condition (i.e., sunny vs. cloudy days) when FS was made in these fields. In comparison, OS was made in controlled indoor environment with stable illumination.

In this study, an existing high-throughput field phenotyping system was used to capture overhead RGB images of soybean field plots at V3–V4 stages when the symptom of IDC was maximized. With two persons, the phenotyping system was able to collect RGB images of all field plots (∼250) in 1 h (**Figure 1B**), much more efficient than the conventional scoring which took 5–6 persons 2–3 h. These images were later displayed on a computer monitor and enabled IDC office scoring by a single researcher. These permanently recorded images had several other advantages. First, they would allow researchers to go back and re-examine them whenever questions arise on the initial score of certain plots; and therefore provide additional quality check of IDC scoring. Second, if these images are accurately benchmarked and annotated for IDC, they can be widely disseminated to test new algorithms for image processing, color feature extraction, and computer-aided IDC rating (Singh et al., 2016). In particular, a large collection of accurately labeled IDC images would enable algorithms such as convolutional neural network to be employed for this purpose (Ubbens and Stavness, 2017).

Our ultimate goal is to have a fully automated system with the pipeline to realize (1) high-quality field RGB image acquisition, (2) onboard image processing and color feature extraction, and (3) real-time IDC scoring with a pre-calibrated statistical prediction model. Toward that end, future research is needed in the following regard. First, image processing algorithm to extract color features from plot images should be widely tested. Algorithms that are robust against variations in illumination, weed pressure and soybean genotypes should be investigated. Second, statistical models that relate color features to IDC FS or OS should be more widely tested. The relationships demonstrated from this study were confined to six fields in Eastern Nebraska and a limited set of soybean genotypes. Before the automated system could be widely adopted, the models need to be tested and validated on sufficiently large environments and soybean genotypes. In addition, the developed pipeline from imaging to automated IDC scoring could be transferred to other phenotyping platforms, such as unmanned aerial vehicles (UAVs) to enable more cost-effective and practical solution to this problem. Finally, color constancy of the images should be investigated in the future experiment, such as including a standard color panel in camera’s field view to enable color calibration.

## Conclusion

The goal of this study was to capture RGB images of soybean plots at V3–V4 stages under the field condition and develop algorithms to realize automated IDC scoring. Two new scoring systems were developed and tested against the conventional field scoring method. First, IDC scoring was performed on the RGB plot images displayed on a computer screen (referred to as OS). Second, computer-based automated scoring was created using image processing and statistical learning (referred to as CS). The following conclusions were drawn.

(1) Higher soil pH and lower soil available iron led to more severe IDC symptoms and lower yield in the six experimental fields.
(2) In the fields with wide ranges of IDC severity, OS and FS were positively correlated; and both of them were negatively correlated with yield.
(3) Color features extracted from soybean plot RGB images predicted IDC FS and OS satisfactorily. The prediction models (automated CS) developed from SVM (overall accuracy > 87%) performed better than those developed from LDA (overall accuracy > 81%).
(4) OS appeared to result in more consistent scoring of IDC severity than FS across the site-years.

## Author Contributions

YG and GG contrived the study. GB, SJ, and WY conducted the experiment and data analysis. YG and GG interpreted the results. GB and SJ drafted the manuscript. YG and GG significantly edited the manuscript.

## Conflict of Interest Statement

The authors declare that the research was conducted in the absence of any commercial or financial relationships that could be construed as a potential conflict of interest.
